# Large Pore Basal Cell Carcinoma: A Case Report

**DOI:** 10.5826/dpc.1103a21

**Published:** 2021-07-08

**Authors:** Ana Inés Lösch, Virginia Mariana González, Félix Alberto Vigovich, Margarita Larralde

**Affiliations:** 1 Dermatology Department, Hospital Alemán, Buenos Aires, Argentina; 2 Histopathology Department, Hospital Alemán, Buenos Aires, Argentina

**Keywords:** basal cell carcinoma, large pore basal cell carcinoma, dermoscopy, pore of Winer, epidermal appendageal tumors

## Case Presentation

**Figure 1:**
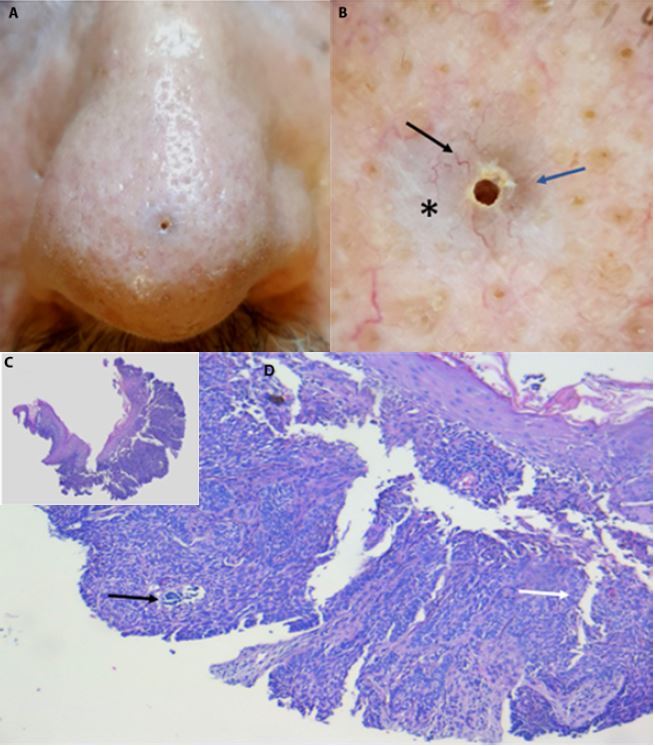
Large pore in the nasal tip. (A) Clinical image. (B) Dermoscopy reveals a large pore surrounded by a whitish pink poorly circumscribed area (*) with superficial branched vessels (black arrow) and grey pigmentation (blue arrow). (C) Histopathology (H&E, ×40). Dilated pilar infundibulum lined with a squamous epithelium. (D) (H&E ×100) Proliferation of basaloid neoplasic cells with stromal retraction sectors (white arrow) underlying the squamous epithelium and focus of calcification (black arrow).

A 75-year-old-male with a history of multiple basal cell carcinomas visited our clinic for aesthetic treatment of a large pore in the nasal tip that he noticed 4 months ago. Following pressure application, keratinous material emerged from the center of the lesion (Figure 1A). Dermoscopy showed a central dilated pore surrounded by a whitish pink poorly circumscribed area with in focus branched vessels. Grey pigmentation and yellowish-white scales were also seen around the central pore (Figure 1B). A punch biopsy was performed and was consistent with large pore basal cell carcinoma (BCC) (Figure 1, C and D). The patient underwent Mohs surgery and plastic reconstruction.

## Teaching Point

Pore-like or large pore basal cell carcinoma is an atypical and very infrequent clinical presentation of BCC. Both dilated pores and BCC, are tumors of follicular origin. Some authors have postulated that the same stem cell could originate them, so they may coexist in a single cutaneous lesion. Dermatologists must take this into account when making differential diagnoses of solitary enlarged pores, especially when localized on the face. Dermoscopy is an excellent tool for its recognition [1, 2].
